# Sulforaphane protects against rotenone-induced neurotoxicity *in vivo*: Involvement of the mTOR, Nrf2, and autophagy pathways

**DOI:** 10.1038/srep32206

**Published:** 2016-08-24

**Authors:** Qian Zhou, Bin Chen, Xindong Wang, Lixin Wu, Yang Yang, Xiaolan Cheng, Zhengli Hu, Xueting Cai, Jie Yang, Xiaoyan Sun, Wuguang Lu, Huaijiang Yan, Jiao Chen, Juan Ye, Jianping Shen, Peng Cao

**Affiliations:** 1 Affiliated Hospital of Integrated Traditional Chinese and Western Medicine, Nanjing University of Chinese Medicine, Nanjing 210028, China; 2 Nanjing Research Institute for Comprehensive Utilization of Wild Plants, Nanjing 210042, China; 3 Laboratory of Cellular and Molecular Biology, Jiangsu Province Academy of Traditional Chinese Medicine, Nanjing 210028, China

## Abstract

Sulforaphane, a naturally occurring compound found in cruciferous vegetables, has been shown to be neuroprotective in several neurological disorders. In this study, we sought to investigate the potential protective effects and associated molecular mechanisms of sulforaphane in an *in vivo* Parkinson’s disease (PD) model, based on rotenone-mediated neurotoxicity. Our results showed that sulforaphane inhibited rotenone-induced locomotor activity deficiency and dopaminergic neuronal loss. Additionally, sulforaphane treatment inhibited the rotenone-induced reactive oxygen species production, malondialdehyde (MDA) accumulation, and resulted in an increased level of total glutathione and reduced glutathione (GSH): oxidized glutathione (GSSG) in the brain. Western blot analysis illustrated that sulforaphane increased the expression of nuclear factor (erythroid-derived 2)-like 2 (Nrf2), heme oxygenase-1 (HO-1), and NAD(P)H quinone oxidoreductase (NQO1), the latter two of which are anti-oxidative enzymes. Moreover, sulforaphane treatment significantly attenuated rotenone-inhibited mTOR-mediated p70S6K and 4E-BP1 signalling pathway, as well as neuronal apoptosis. In addition, sulforaphane rescued rotenone-inhibited autophagy, as detected by LC3-II. Collectively, these findings demonstrated that sulforaphane exert neuroprotective effect involving Nrf2-dependent reductions in oxidative stress, mTOR-dependent inhibition of neuronal apoptosis, and the restoration of normal autophagy. Sulforaphane appears to be a promising compound with neuroprotective properties that may play an important role in preventing PD.

Parkinson’s disease (PD) is a chronically progressive disease, that most commonly manifests between the fifth and seventh decade with resting tremor on one or both sides, bradykinesia, rigidity, and abnormal postural reflexes^1^. It affects approximately 6 million people worldwide, with the prevalence expected to double over the next two decades in parallel with an increasingly aged population^2^. Its pathological hallmarks include loss of dopaminergic neurons in specific brain regions, and the presence of proteinaceous cytoplasmic inclusions known as Lewy bodies in the remaining dopaminergic neurons^3^. Although PD has been studied intensively for almost two centuries, since James Parkinson gave the first description of the disease in 1817^4^, the aetiology and pathogenesis of the disease remain unknown. Currently, there is no cure for PD, and additional effective treatments for this devastating disease are urgently needed.

Oxidative stress is widely considered to be central to many forms of PD^5^. Postmortem studies have revealed evidence for oxidative injury in PD brains, demonstrating lipid peroxidation and oxidative modification of proteins and DNA^6^. The oxidative stress results from an imbalance of pro-oxidant/antioxidant homeostasis that leads to an abnormal production of reactive oxygen species (ROS). The brain is particularly vulnerable to oxidative stress because of its high oxygen consumption, high content of oxidizable polyunsaturated fatty acids, and low antioxidant defence capacities; this is especially true for aging brains^7^. NF-E2-related factor-2 (Nrf2) is a transcription factor that regulates the expression of antioxidant and phase II detoxifying enzymes. One critical enzyme induced by Nrf2 is NAD(P)H quinone oxidoreductase-1 (NQO1), which detoxifies protein-bound quinone, and maintains alpha-tocopherol and coenzyme Q10 in their reduced (antioxidant) states. Another Nrf2-regulated protein is the stress responsive inducible enzyme heme oxygenase 1 (HO-1), which metabolizes prooxidant heme to the antioxidant pigment biliverdin, ferrous iron, and carbon monoxide^8^. The Nrf2 pathway responds to ROS by activating the transcription of phase II detoxification enzymes. Thus, activation of Nrf2 appears to be an attractive approach for treating or mitigating PD^9^.

Mammalian target of rapamycin (mTOR), a serine/threonine (Ser/Thr) protein kinase, is a central controller of cell proliferation, growth, and survival. mTOR regulates phosphorylation of p70 S6 kinase (p70S6K) and eukaryotic initiation factor 4E (eIF4E) binding protein 1 (4E-BP1), the two best-characterized downstream effector molecules of mTOR^10^. Studies have demonstrated that loss of mTOR activity may be detrimental during PD, as mTOR dysfunction has been shown in animal models of PD to lead to the death/apoptosis of dopaminergic neurons^11^,^12^. Depression of mTOR signalling by siRNA interference inhibits phosphorylation of both p70S6K and 4E-BP1, which leads to apoptosis^13^. However, dopaminergic neuronal death/apoptosis can be blocked with agents that increase mTOR activity^14^. Recently, our group has demonstrated that PD mimetics including 6-hydroxydopamine (6-OHDA), N-methyl-4-phenylpridine (MPP^+^), and rotenone suppressed mTOR signalling, accelerated cleavage of caspase-3 and PARP in PC12 cells and primary neurons, and subsequently triggered neuronal apoptosis. However, restoration of mTOR, p70S6K, or 4E-BP1 was remarkably potent in protecting against neuronal apoptosis caused by these PD mimetics^10^,^15^.

Studies have now confirmed that dysregulation of autophagy results in the accumulation of pathologic proteins and/or damaged organelles, as is commonly observed in PD^16^. In particular, autophagy is deregulated in PD brains^17^. Many studies have consistently shown that rapamycin is neuroprotective against PD through autophagy enhancement^18^,^19^. Microtubule-associated protein light chain 3 (LC3) is one of the most commonly used biomarkers for autophagy process. LC3-I is cytosolic, whereas LC3-II is membrane bound. During autophagy, LC3-I is lipidated by an ubiquitin-like system to generate LC3-II^20^. Thus, the amount of LC3-II is correlated with the autophagy level, and is a reliable marker for monitoring autophagy-related processes^21^.

Sulforaphane, a naturally occurring compound found in cruciferous vegetables (i.e. broccoli, cabbage, watercress and Brussels sprouts), has been reported to have potential as a therapeutic agent or preventative in PD. For example, sulforaphane was shown *in vitro* to be cytoprotective against damage evoked by 6-OHDA, MPP^+^, and rotenone^22^,^23^,^24^,^25^,^26^. Using *in vivo* neurodegeneration models, Jazwa *et al.* demonstrated that sulforaphane, in an Nrf2-dependent manner, protected against nigral dopaminergic cell death, astrogliosis, and microgliosis in the 1-methyl-4-phenyl-1,2,3,6-tetrahydropyridine (MPTP) subacute mouse model of PD^27^. Morroni *et al.* reported that sulforaphane exerted a neuroprotective effect in a 6-OHDA-induced acute mouse model, and attributed it to the ability of sulforaphane to enhance glutathione levels and its dependent enzymes, to up-regulate Nrf2, and to modulate the ERK1/2 pathway in the brain^28^. Surprisingly, no data regarding the effectiveness of sulforaphane in a rotenone-induced chronic PD model have been reported.

Rotenone, derived from the roots or leaves of certain plant species, is a pesticide that is widely used to kill insects and nuisance fish in lakes around the world^5^. Rotenone is extremely lipophilic, and thus freely crosses cellular membranes independently of any transporters (unlike MPP^+^); it easily crosses the blood-brain barrier and enters into the brain rapidly^29^. Unlike 6-OHDA-based models, generating the chronic oral rotenone model in mice does not require stereotaxic neurosurgical intervention. In fact, accumulated data show rotenone exposure to humans would most commonly involve ingestion^1^. Interestingly, chronic oral administration of rotenone to rodents *in vivo* induces many key features of PD, including selectively and progressive nigrostriatal DA neurodegeneration, abnormal behaviours (not seen in the MPTP mouse model), and formation of Lewy bodies (not seen in either the MPTP or 6-OHDA mouse model)^30^. In this study, by using chronic oral rotenone treatment as an *in vivo* PD model, we explored the effectiveness of sulforaphane against rotenone-induced motor deficits and dopaminergic neuron loss; we also determined whether the possible underlying mechanisms of its effects were associated with Nrf2-regulated anti-oxidative, mTOR-mediated anti-apoptotic, and/or autophagy systems.

## Material and Methods

### Chemicals and reagents

Rotenone, sulforaphane, dimethyl sulfoxide (DMSO), 2′,7′-dichlorodihydrofluorescein diacetate (DCFH-DA) and 2′,7′-dichlorofluorescein (DCF) were purchased from Sigma (St. Louis, MO, USA); CMC: (Nacalai Tesque, Kyoto, Japan); Protease and phosphatase inhibitor mini tables (Thermo Scientific, USA); RIPA lysis buffer was purchased from Beyotime, China. The following antibodies were used: phospho-S6K (Thr389), 4E-BP1, Cleaved-caspase-3, LC3A/B (Cell Signalling Technology, Beverly, MA, USA); phospho-mTOR (Ser2448), mTOR (all from sigma); p70S6K; Nrf2, heme oxygenase-1 (HO-1); NAD(P)H dehydrogenase, quinone 1/(NQO1) and monoclonal mouse anti-glyceraldehyde-3-phosphate dehydrogenase (GAPDH) (Santa Cruz Biotechnology, Santa Cruz, CA, USA). Tyrosine hydroxylase (TH) (Millipore, Billerica, MA). Dulbecco’s Modified Eagle’s Medium (GIBCO, Gaithersburg, MD, USA). Other chemicals were provided by local commercial sources and were of analytical grade quality.

### Animals care

All experimental procedures were approved by the Institutional Animal Care and Use Committee of the Jiangsu Province Institute of Traditional Chinese Medicine and written up following the ARRIVE guidelines. Experiments were performed in strict accordance with the National Institutes of Health (NIH) Guidelines for the Care and Use of Laboratory Animals. Male C57BL/6 mice (7–8 weeks old, 25–28 g) were purchased from the Institute of Laboratory Animal Science at the Chinese Academy of Medical Sciences (Beijing, China) and housed in a specific pathogen-free facility maintained at 20–22 °C, with cycles of 12 h light-dark and food and water *ad libitum*. All mice were initially allowed to adapt to the experimental animal laboratory for 1 week.

### Cell culture

SH-SY5Y cells were grown in Dulbecco’s Modified Eagle’s Medium (DMEM) supplemented with 10% heat-inactivated fetal bovine serum, and cultured at 37 °C under humidified 5% CO_2_ atmosphere.

### Experimental design

**Figure 1:**
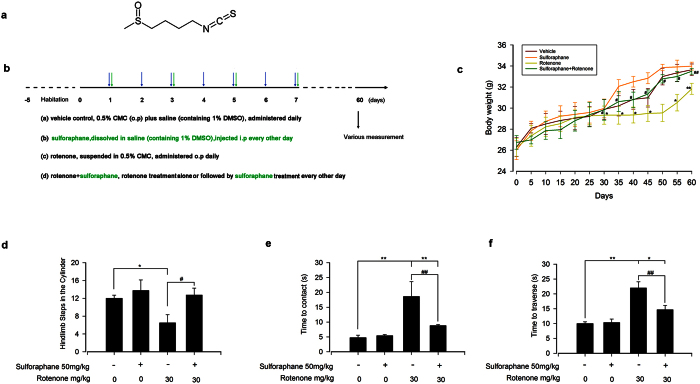
Sulforaphane ameliorated rotenone-induced motor function deficits in mice. (**a**) Chemical structure of sulforaphane. Animals were submitted to the combined protocol of sulforaphane and rotenone (**b**). Body weights were measured every five days for the full 60 days (**c**). Animals underwent behavioural tests on day 60. Spontaneous activity in the cylinder (**d**), adhesive removal test (**e**) and challenging beam (**f**) were measured. Each value is presented as mean ± SEM, n = 3. *P < 0.05, **P < 0.01 vs. control group; ^#^P < 0.05, ^##^P < 0.01 rotenone group vs. rotenone + sulforaphane treatment group.

Sulforaphane stock solution was prepared using dimethyl sulfoxide (DMSO) and stored at −20 °C. Immediately prior to experimentation, the stock solution was diluted with sterile saline to a concentration of 50 mg/kg (final DMSO concentration 1%)^31^,^32^. The pharmacological effects *in vivo* were examined using the following groups (n = 10 per group): (a) vehicle control, 0.5% carboxymethyl cellulose sodium salt (CMC) plus saline (containing 1% DMSO) administered once daily; (b) sulforaphane (50 mg/kg dissolved in saline (containing 1% DMSO)) injected intraperitoneally every other day; (c) rotenone (30 mg/kg rotenone suspended in 0.5% CMC) administered orally daily for 60 consecutive days; and (d) rotenone + sulforaphane, rotenone treatment orally alone or followed by sulforaphane injected intraperitoneally every other day. Body weight was measured every five days for the 60-day duration, while the mice were undergoing behaviour tests. Fig. 1b shows the experimental schedules.

### Behavioural Tests

The tests below have been validated in PD mouse models^33^. In each test, mice were tested individually, and the equipment cleaned between each animal (except when tests utilised home cages).

### Spontaneous activity tests

A mouse was placed in a clear glass cylinder and recorded with a video camera for 3 min in a quiet area, to prevent distractions and accidentally causing freezing behaviour. The videos were used to manually count the number of rears per mouse. A rear is defined as a vertical movement with both forelimbs above shoulder level and making contact with the cylinder wall, followed by removal of both forelimbs from the cylinder wall and contact with the floor^34^. All mice performed 3 trials.

### Adhesive Removal

A mouse was kept in its home cage, but the feeder bin removed to allow the mouse to habituate to the testing environment for 1 hour. Then the mouse was gently restrained, and a pair of small forceps used to place one adhesive label onto its snout. The mouse was released, and a stopwatch used to record the time until the attempts to contact the label with its forepaws, and the time to remove the label (contact and removal time, respectively). The mice were given a maximum of two minutes. All mice performed 3 trials.

### Challenging Beam Traversals

This evaluates sensorimotor integration by requiring a mouse to balance steadily on a narrow beam. The test-apparatus consists of four 25 cm beam sections of different widths placed on top of inverted mouse cages. A mouse was trained by being positioned on the widest section of the beam, and then trained to step forward on the beam to return to its home cage. The training was performed two consecutive days, until the mouse traversed the full beam on its own without correction. On the test day, a video camera was placed nearby to record the beam travel times. All mice performed 3 trials.

### Tissue Preparation

After the behaviour tests, the mouse was euthanized by decapitation. The whole brain, cerebral cortex, and striatum were dissected carefully on dry ice as described previously^35^. The whole brain regions were immediately homogenised for ROS, malondialdehyde (MDA), total glutathione and reduced glutathione (GSH): oxidized glutathione (GSSG) ratio assays. Some of the dissected cerebral cortex and striatum were quickly immersion-perfused with 4% paraformaldehyde in 0.1 M phosphate-buffered saline (PBS) for 2 days, and then transferred to 15% sucrose solution in PBS containing 0.1% sodium azide at 4 °C. The other non-perfused brain regions were snap frozen in −80 °C 2-methylbutane and stored in a −80 °C freezer until further processing for protein extraction.

### ROS Assay

ROS generation was measured based on the oxidation of 2′7′-dichlorodihydrofluorescein diacetate (DCFH-DA) to 2′7′-dichlorodihydrofluorescein diacetate (DCF)^36^. Briefly, the brain homogenate was diluted 1:20 (v:v) with ice-cold Locke’s buffer (154 mM NaCcl, 5.6 mM KCl, 3.6 mM NaHCO3, 2.0 mM CaCl2, 10 mM D-glucose and 5 mM HEPES, pH 7.4) to obtain a concentration of 10 mg tissue/ml. The reaction mixture (1 ml) containing Locke’s buffer (pH 7.4), 0.2 ml homogenate and 10 μl of DCFH-DA (5 mM) was incubated for 45 min at room temperature, and the fluorescence intensity of DCF was recorded by excitation at 485 nm and emission at 535 nm using a Synergy^TM^ 2 Multi-function Microplate Reader (Bio-Tek Instruments Inc., Winooski, Vermont, USA). Background fluorescence was corrected by the inclusion of parallel blanks. Total protein concentration was determined using the bicinchoninic acid assay kit (Pierce, Rockford, IL, USA). ROS formation was quantified from a DCF-standard curve and data are expressed as pmol DCF formed/min/mg protein.

### MDA Assay

This assay assesses lipid peroxidation^29^. Brain tissue was homogenised with 1:10 (w/v) PBS on ice, then centrifuged at 3,000 rpm for 15 min at 4 °C in an Eppendorf centrifuge, and the supernatant was collected. Protein concentration in the supernatant was determined using the bicinchoninic acid assay kit (Pierce, Rockford, IL, USA). The concentration of MDA was measured and expressed as nmol/mg protein using an MDA assay kit (Jiancheng Institute of Biotechnology, Nanjing, China), according to the manufacturer’s protocols.

### Total Glutathione Levels and GSH:GSSG Ratio

Total glutathione and GSSG were determined using a GSH and GSSG Assay Kit (Beyotime Institute of Biotechnology, Shanghai, China) according to the manufacturer’s protocol. Absorbance was measured at 412 nm using a Biotek microplate reader. GSH was calculated as the difference between total glutathione and GSSG, and GSH:GSSG ratio was determined. Protein concentration was determined using the bicinchoninic acid assay kit (Pierce, Rockford, IL, USA). Total glutathione content is expressed as nmol/mg protein.

### Immunohistochemistry Analysis

The cryoprotected cerebral cortex and striatum were cut into 30 μM slices on a cryostat as previously described^27^. Sections were rinsed in Tris-buffered saline (TBS). Tissue peroxidase was inactivated by incubating in 3% hydrogen peroxide in TBS for 30 min. After three washes in PBS, sections were incubated for 3 h in blocking solution (3% bovine serum albumin (BSA), 0.3% Triton X-100 in TBS) and then for 24 h at 4 °C with mouse monoclonal antibodies against tyrosine hydroxylase (TH) diluted in PBS containing 1% BSA. Sections were then rinsed in PBS and incubated with the appropriate biotinylated secondary antibody at 1/1000 dilution for 1 h at room temperature. Sections were subsequently developed using the avidin-biotin peroxidase complex system (ABC kit, BOSTER Ltd. Wuhan) followed by counterstaining with haematoxylin. The images of stained sections were taken with a Nikon Eclipse Ti-S microscope equipped with a digital camera. Densitometric quantification of TH-positive dopaminergic neurons in the striatum and fibres in the cerebral cortex was performed using Image-Pro Plus 6.0 software (Media Cybernetics Inc., Newburyport, MA).

### Western Blotting Assay

For protein electrophoresis and immunoblotting, the cerebral cortex and striatum were homogenized on ice with lysis buffer (Beyotime Institute of Biotechnology, Shanghai, China). Protease and phosphatase inhibitor min-tablets (Thermo Scientific, USA) were added according to the manufacturer’s instructions. After centrifugation, protein extracts were collected, and 50 μg of protein lysates were resolved by SDS-PAGE and electrophoretically transferred onto Immobilon-P membranes (0.22 μM; Millipore). Membranes were blocked with 5% non-fat milk for 2 h, then incubated with primary antibodies overnight at 4 °C. After washing with PBS-T, membranes were incubated with the fluorescent-conjugated secondary antibodies in PBS-T/milk for 2 h at room temperature. Membranes were then rewashed, after which the labelled proteins were detected using an Odyssey infrared fluorescent scanner (LI-COR) with Odyssey ver. 3 software used for analysis.

### Quantification and statistics

Results were expressed as mean ± standard error of the mean (mean ± SEM). Student’s t-test for non-paired replicates was used to identify statistically significant differences between treatment means. Group variability and interaction were compared using either one-way or two-way ANOVA followed by Bonferroni’s post-tests to compare replicates means. A level of P < 0.05 was considered to be significant.

## Results

### Sulforaphane ameliorated rotenone-induced motor function deficits in mice

Sulforaphane did not show any toxicity in our study, and has been used previously as a protective reagent at a dose of 50 mg/kg *in vivo*^27^,^37^,^38^. We used the schematic design depicted in Fig. 1b. Body weight gain was significantly attenuated from day 30 to day 60 in the rotenone-exposed group, compared with the vehicle-treated group. Sulforaphane treatment did not affect body weight gain, but significantly rescued the reduction of weight gain induced by rotenone (Fig. 1c).

Two hours after administration of the last dose of drugs, mice were observed for sensorimotor function changes. We found no difference in neurobehavioral performance between the sulforaphane and the vehicle group (Fig. 1d–f), whereas, the rotenone group showed a robust decrease in hind limb stepping in the cylinder (Fig. 1d). At the same line, the time taken by the rotenone group was significantly longer in the adhesive removal and challenging beam traversal tests (Fig. 1e,f). However, intraperitoneal administration of sulforaphane at 50 mg/kg every other day before rotenone exposure significantly rescued motor function (Fig. 1d,f). These results suggest that sulforaphane can reverse the loss of body weight gain and PD-related motor deficits caused by rotenone.

### Sulforaphane attenuated rotenone-induced dopaminergic neurodegeneration

**Figure 2:**
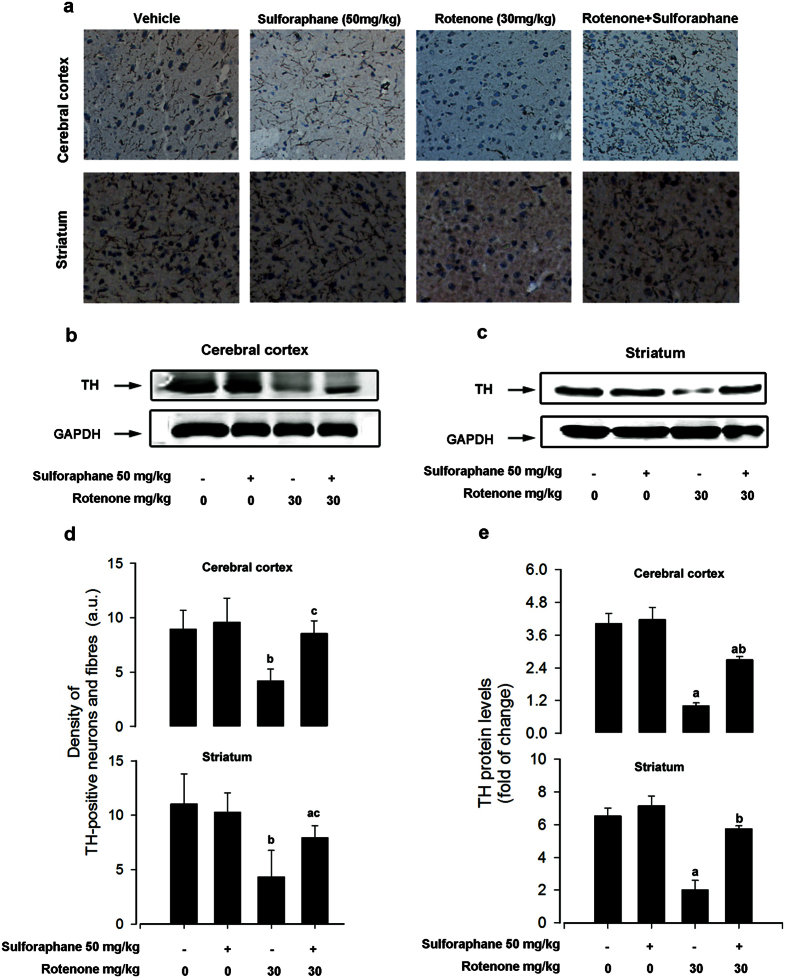
Sulforaphane attenuated rotenone-induced dopaminergic neurodegeneration. (**a**) Immunohistochemistry with anti-tyrosine hydroxylase (TH) antibody in the brain. Pictures show 30 μM-thick sections of cerebral cortex and striatum between different groups. (**b**) Immunoblots of cerebral cortex protein extracts blotted with TH and GAPDH antibodies. (**c**) Immunoblots of striatal protein extracts blotted with TH and GAPDH antibodies. (**d**) Densitometric quantification of TH-positive neurons and fibres. (**e**) Blots for TH were semi-quantified using NIH ImageJ. Relative density expressed as the ratio TH/GAPDH. Each value is presented as mean ± SEM, n = 3. ^a^P < 0.01 vs. control group; ^b^P < 0.05, ^c^P < 0.01 rotenone group vs. rotenone + sulforaphane treatment group.

Since the pathological changes in PD include a loss of dopaminergic neurons (TH-positive neurons), and a decrease in TH expression on the lesioned side, we measured the integrated intensity of labelling in the cerebral cortex and striatum. After 60 days of rotenone exposure, a marked loss of TH-positive neurons and fibres were observed (Fig. 2a). The optical density was decreased in TH-positive neurons by 60% in the stratum, and TH-stained fibres, by 53% in the cerebral cortex, respectively (Fig. 2d). Interestingly, co-administration of sulforaphane significantly rescued the rotenone-induced the loss of TH-stained neurons and fibres (Fig. 2a,d). To further confirm the effects of sulforaphane in neuroprotection after rotenone injury, the TH protein levels were analysed by western blotting. Rotenone elicited a 75% and a 55% reduction of TH expression in the extracted cerebral cortex and striatum (Fig. 2b,c). More importantly, sulforaphane rescued TH protein levels, to over 50% of vehicle control group levels, in both lesioned brain regions (Fig. 2e). These results indicate that sulforaphane attenuated rotenone-induced dopaminergic neurodegeneration.

### Sulforaphane rescued rotenone-induced oxidative damage by activating the Nrf2 pathway

**Figure 3:**
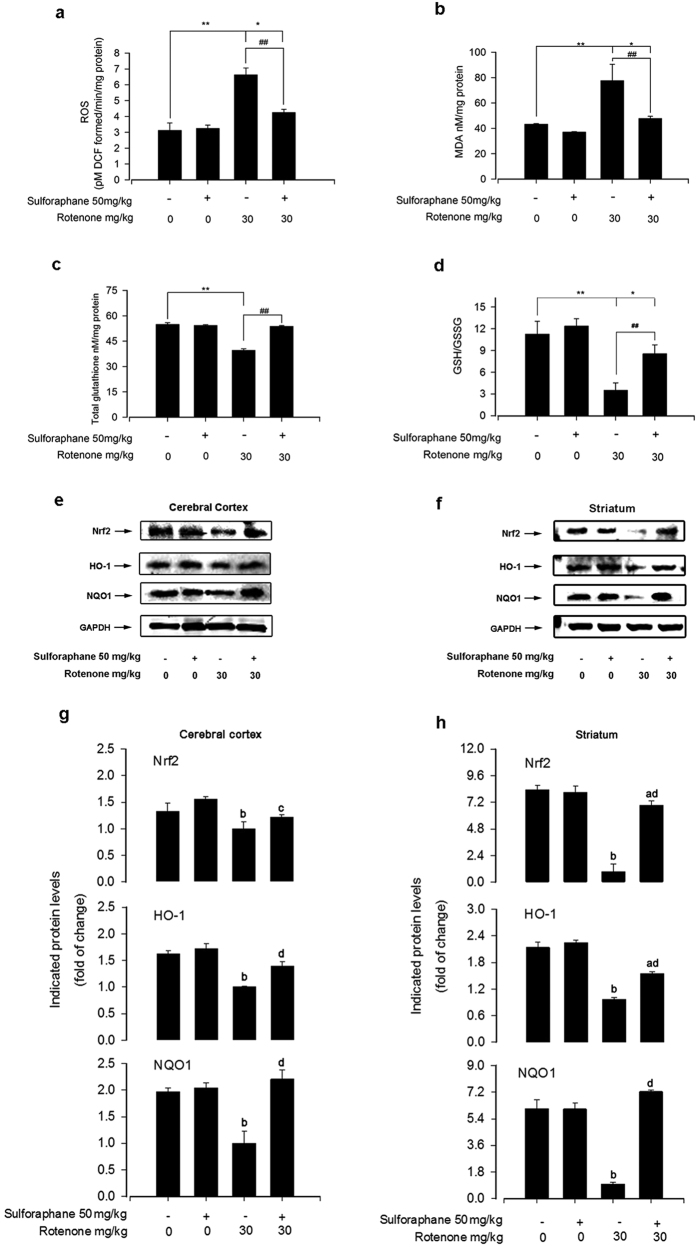
Sulforaphane rescued rotenone-induced oxidative damage by activating the Nrf2 pathway. (**a**) Reactive oxygen species (ROS) level was measured based on the oxidation of DCFH-DA to DCF using a microplate reader. The malondialdehyde (MDA) levels (**b**), total glutathione activity (**c**) and reduced:oxidized glutathione (GSH/GSSG) ratio (**d**) in the brain. GAPDH was used as loading control for western blots. At least three independent experiments were performed. Representative immunoblots for Nrf2, HO-1, NQO1 and GAPDH in cerebral cortex (**e**) and striatum (**f**). Blots for Nrf2, HO-1, and NQO1 were semi-quantified using NIH imageJ. Relative density is expressed as the ratio Nrf2/GAPDH, HO-1/GAPDH, NQO1/GAPDH in cerebral cortex (**g**) and striatum (**h**), respectively. Each value is presented as mean ± SEM, n = 3. ^a^P < 0.05, ^b^P < 0.01 vs. control group; ^c^P < 0.05, ^d^P < 0.01 rotenone group vs. rotenone + sulforaphane treatment group.

ROS generation is a necessary by-product of oxidative metabolism, and is tightly regulated in cells by various oxidant and antioxidant enzymes^39^. MDA is a product of lipid peroxidation and a good indicator of oxidative stress in a cell or tissue^29^. We firstly investigated the effects of sulforaphane on rotenone-induced ROS production and MDA levels. Results indicated rotenone induced robust ROS generation (Fig. 3a). In addition, exposure of mice to 30 mg/kg rotenone for 60 day significantly increased MDA levels (Fig. 3b). Rotenone also resulted in a consistent, significant decrease in total glutathione content (Fig. 3c) and GSH:GSSG ratio (Fig. 3d). However, co-administration of sulforaphane potently inhibited all of the above pro-oxidative stress effects (Fig. 3a–d). These results imply that sulforaphane may be able to prevent oxidative damage in the brain after rotenone exposure.

It is well known that the Nrf2 pathway is able regulate antioxidant enzymes, especially HO-1 and NQO1^3^,^8^. To explore the molecular mechanisms underlying the protective effects of sulforaphane in the rotenone mouse model of PD, we next analysed Nrf2 pathway in both cerebral cortex and striatum. Western blot analysis demonstrated that exposure to rotenone for 60 days resulted in a strong inhibition of Nrf2 expression, which was profoundly restored by co-administration of sulforaphane (50 mg/kg), in the cerebral cortex and striatum, respectively (Fig. 3e,f). We also analysed the expression of Nrf2-dependent anti-oxidative and detoxifying enzymes after sulforaphane treatment in the rotenone model mice. Compared with the rotenone group, the sulforaphane + rotenone group demonstrated increased protein expression of HO-1, 1.4-fold higher in the cerebral cortex and 1.6-fold higher in the striatum, respectively (Fig. 3e–h). Similarly, sulforaphane partially rescued rotenone-decreased expression of NQO1 level in the cerebral cortex, improving expression by 2.2-fold, and striatum, by 7.3-fold (Fig. 3e–h). Collectively, treatment with sulforaphane rescued rotenone induced oxidative damage by activating the Nrf2 pathway.

### Sulforaphane inhibited rotenone-induced neuronal apoptosis by restoring the mTOR pathway

**Figure 4:**
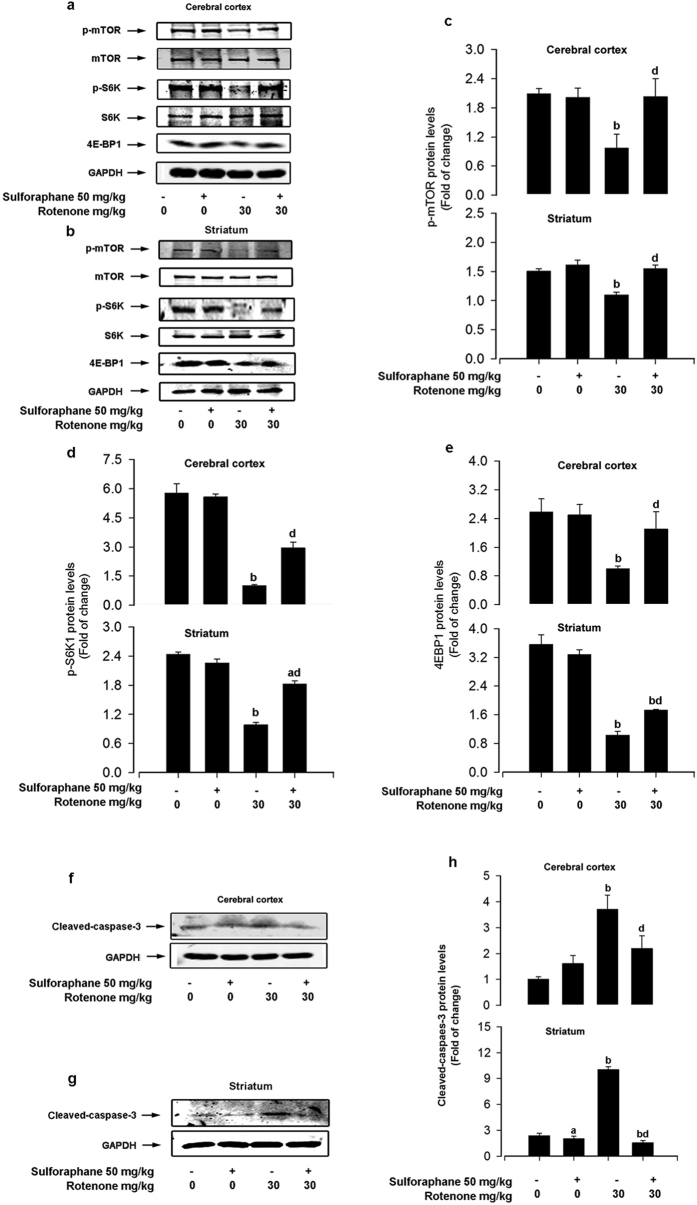
Sulforaphane inhibited rotenone-induced neuronal apoptosis by restoring mTOR pathway activity. Representative immunoblots for p-mTOR, mTOR, p-p70S6K, p-70S6K, 4E-BP1, and GAPDH in cerebral cortex (**a**) and striatum (**b**). Blots for p-mTOR, p-p70S6K and 4E-BP1 were semi-quantified using NIH imageJ. Relative density expressed as the ratios of p-mTOR/GAPDH (**c**), p-p70S6K/GAPDH (**d**), and 4E-BP1/GAPDH (**e**) in cerebral cortex and striatum, respectively. Representative immunoblots for cleaved-caspase-3 in cerebral cortex (**f**) and striatum (**g**). Blots for cleaved-caspase-3 were semi-quantified using NIH imageJ. Relative density expressed as the ratio cleaved-caspase-3/GAPDH (**h**). Each value is presented as mean ± SEM, n = 3. ^a^P < 0.05, ^b^P < 0.01 vs. control group; ^c^P < 0.05, ^d^P < 0.01 rotenone group vs. rotenone + sulforaphane treatment group.

Disturbances in the balance of mTOR activity in the brain can impair neuronal functions, and have detrimental consequences on neuronal regeneration after damage^40^. p70S6K and 4E-BP1 are the two best-characterized downstream effector molecules of mTOR^10^. In the nervous system, inhibition of mTOR pathway, including its downstream effectors p70S6K and 4E-BP1, usually promotes neuronal apoptosis^11^. Previously, we found that rotenone could negatively regulate mTOR-mediated p70S6K and 4E-BP1/Eif4E pathways, that leads to caspase-3 dependent neuronal apoptosis *in vitro*^15^. Here, we tested the hypothesis that sulforaphane prevents rotenone-induced neuronal apoptosis by targeting the mTOR pathway *in vivo*. Western blot analysis revealed that sulforaphane alone did not obviously alter the expression of mTOR signalling components, but was highly resistant to the inhibition of phosphorylation of mTOR, p70S6K and 4E-BP1 by rotenone, in both the cerebral cortex and striatum (Fig. 4a,b).

To further assess the role of mTOR in neuronal survival and confirm the apoptosis signalling pathways regulated by sulforaphane, we analysed the level of cleaved-caspase-3. As shown in Fig. 4f,g, sulforaphane alone was not toxic to brain tissues. By contrast, the rotenone group demonstrated increased protein expression of cleaved-caspase-3 relative to the control group. In addition, co-administration of sulforaphane potently blocked rotenone-increased expression of cleaved-caspase-3 (Fig. 4f,g). Collectively, our findings support the notion that sulforaphane inhibited rotenone-induced neuronal apoptosis by restoring mTOR pathway.

### Sulforaphane protected against rotenone neurotoxicity via modulating autophagy

**Figure 5:**
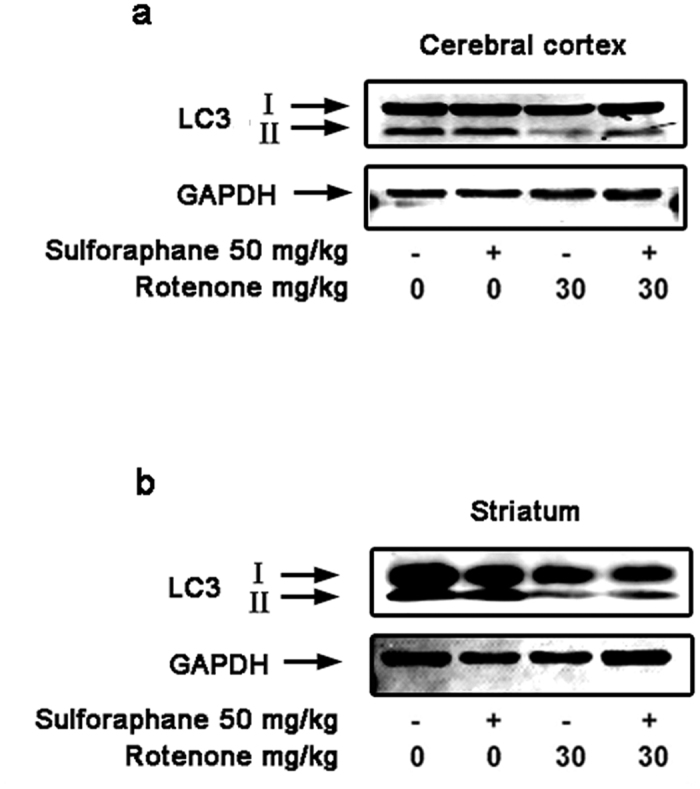
Sulforaphane protected against rotenone neurotoxicity via modulating autophagy. Representative immunoblots for LC3 and GAPDH in cerebral cortex (**a**) and in striatum (**b**).

Recent reports indicate that the pathology of PD is closely related to the accumulation of aberrant proteins, which in theory could be reduced by autophagy^16^. Therefore, we evaluated whether sulforaphane protected against rotenone neurotoxicity by modulating autophagy using the LC3-II biomarker (see Introduction). In the rotenone group, LC3-II expression was significantly decreased compared to the vehicle-injected control. Though sulforaphane alone did not increase LC3-II levels, it blocked the significant decrease in LC3-II expression when co-administered with rotenone *in vivo*. (Fig. 5a,b). We further verified these results *in vitro*. SH-SY5Y cell line cultures were pre-treated with sulforaphane (10 μM) for 2 h and then exposed to rotenone (0.5 and 1 μM) for 24 h. Consistent with the observation *in vivo*, sulforaphane alone did not affect LC3-II expression, but significantly rescued rotenone-inhibited LC3-II expression. In addition, sulforaphane inhibited rotenone-induced neuronal apoptosis, as detected by the protein expression of cleaved-caspase-3 in the SH-SY5Y cell line (Fig. S1).

## Discussion

PD is a commonly occurring neurodegenerative disorder that produces muscular rigidity, bradykinesia, tremor in resting limbs and loss of postural balance^1^,^39^. The basic neuropathology of PD involves the selective degeneration of dopaminergic cells in specific brain regions like the striatum; when degeneration in these neurons reaches a threshold reduction of 80% dopamine, the motor symptoms of PD emerge^29^. The cerebral cortex, which is abundant with neurons and easily accessible, demonstrates its importance in modelling PD^3^.

The plant-based pesticide rotenone is a prototypical toxin that can mimic clinical and pathological features of PD in an animal model^5^. We successfully utilized the rotenone-based model established by Inden *et al.*^30^, inducing PD-like symptoms characterised by evident motor deficits and the loss of TH-positive neurons in mice (Figs 1 and  2). Sulforaphane, derived from the corresponding glucoraphanin glucosinolate found in abundance in cruciferous vegetables, has recently been recognised as an effective neuroprotective compound. Tarozzi *et al.* found that pre-treatment with sulforaphane effectively abolished 6-OHDA and H_2_O_2_ induced oxidative stress and cytotoxicity in a dopaminergic-like neuroblastoma SH-SY5Y cell line^24^. Denzer *et al.* indicated that sulforaphane was protective effect against rotenone-induced cell damage in PC12 cells^26^. In addition, sulforaphane crosses the blood brain barrier, and was neuroprotective in the MPTP and 6-OHDA mouse model of PD^27^,^28^. Consistent with the above results, we found that sulforaphane also effectively protected against rotenone-induced motor dysfunction and TH–positive neurons loss *in vivo* (Figs 1 and 2).

Patients with PD exhibit prominent evidence of oxidative stress, including decreased levels of reduced glutathione and increased oxidative modification to DNA, lipids, and protein^5^. Recently, we showed that rotenone elicited ROS overproduction in PC12 cell line and primary neurons, which led to neurotoxicity^15^. In this study, long-term administration of rotenone in C57BL/6 mice triggered marked oxidative stress in the brain. However, pre-treatment with sulforaphane markedly inhibited rotenone-mediated increases in every oxidative marker we tested (Fig. 3a–d). These results are in line with a recent study demonstrating that sulforaphane decreased dopaminergic cell loss, as well as enhanced GSH synthesis and conjugation, in both *in vitro* and *in vivo* drosophila models of PD^41^. In addition, the reduced total glutathione content and lower ratio of GSH:GSSG were analysed and interpreted as evidence of redox imbalance, and has been associated with various human disease, including PD^42^,^43^. Several important findings suggest Nrf2 activity declines is a significant risk factor for PD^27^. Similarly, rotenone triggered a robust decrease of Nrf2, HO-1 and NQO1 expression in the cerebral cortex and striatum, which was potently rescued by sulforaphane (Fig. 3e–h). Some studies suggested that sulforaphane treatment alone could induce Nrf2 and its dependent enzymes *in vivo*^44^,^45^. However, in our experiment, even a 2-month repeated injection protocol did not appear to activate the Nrf2 pathway in healthy animals. Interestingly, our results are consistent with other studies showing that repeated sulforaphane treatment could result in a long-term elevation of free radical defence, though the sustained elevation of expression of Nrf2, HO-1 and NQO1 seems unlikely^27^,^46^,^47^. Collectively, our data support the notion that sulforaphane protects against rotenone-induced oxidative stress by activating the Nrf2 pathway.

Neuronal apoptosis is reported to play a predominant role in the pathological progress of neuron loss in PD^48^. There are two major pathways through which apoptosis are induced, but both pathways converge on caspase-3 activation, resulting in nuclear degradation and cellular morphological change^49^. mTOR signalling is involved in regulating differentiation and survival in neurons, as well as synaptic plasticity, learning and memory, and food uptake in adult brain^15^. In the nervous system, inhibition of mTOR-mediated p70S6K and 4E-BP1 signalling usually promotes neuronal apoptosis^11^. Rotenone is assumed to exert its cytotoxicity in part through the induction of apoptosis. Previously, we have found that by inhibiting mTOR signalling, rotenone increased cleaved-caspase-3 expression and led to neuronal apoptosis *in vitro*^10^,^15^. We also demonstrated that ectopic expression of wild-type mTOR, constitutively activate S6K1, or downregulated 4E-BP1 partially prevented rotenone-induced neuronal apoptosis, suggesting that rotenone-induced neuronal loss in PD may be prevented by activating mTOR signalling^15^. Consistent with these data, we found that rotenone inhibited mTOR-mediated p70S6K and 4E-BP1 activity, and triggered neuronal apoptosis, as detected by increased expression of cleaved-caspase-3 *in vivo* (Fig. 4). Studies have shown that sulforaphane is able to regulate cell cycle arrest and apoptosis^50^. For example, sulforaphane attenuated cell necrosis and apoptosis in renal injury rats^51^ and type 1 diabetes mice^52^ models. Interestingly, it is reported that sulforaphane activated the PI3K/Akt pathway to confer protection against 6-OHDA induction of apoptosis in PC12 cells^23^. Here, as expected, co-treatment with sulforaphane strongly reversed rotenone-inhibited phosphorylation of mTOR, p70S6K and 4EBP1 (Fig. 4a–e), as well as neuronal apoptosis detected by cleaved-caspase-3 (Fig. 4f–h). Our findings strongly suggest that sulforaphane attenuated rotenone-induced inhibition of mTOR signalling pathway as well as neuronal apoptosis.

Autophagy has been reported to be involved in rotenone-mediated neurotoxicity, based on LC3-II decreases^53^. Our results herein showed that rotenone treatment triggered a robust decrease of LC3-II, probably through inhibition of autophagy, which aggravated deposition of aberrant proteins and subsequent cell death^53^. Sulforaphane alone cannot directly stimulate autophagy at the 50 mg/kg dose associated with neuroprotection, but it could rescue rotenone-inhibited autophagy (Fig. 5a,b). It seems this study is in contrast to recent reports that indicate that administration of sulforaphane alone enhances autophagic activities^54^,^55^. To address this discrepancy, we further investigated the effects of sulforaphane and rotenone treatments in the SH-SY5Y cell line. Our results were in line with the *in vivo* data, that sulforaphane itself did not affect LC3-II expression, but significantly rescued the rotenone-inhibited expression of the protein. Furthermore, sulforaphane significantly attenuated rotenone-induced neuronal apoptosis in the SH-SY5Y cell line (Fig. S1). Our observations imply that sulforaphane may not directly affect autophagy at the concentration and over the time course associated with neuroprotection that we used, but it can still rescue rotenone-inhibited autophagy. This is consistent with previous investigations showing that sulforaphane induction of autophagy is cell-type, concentration and time dependent^54^,^56^.

**Figure 6:**
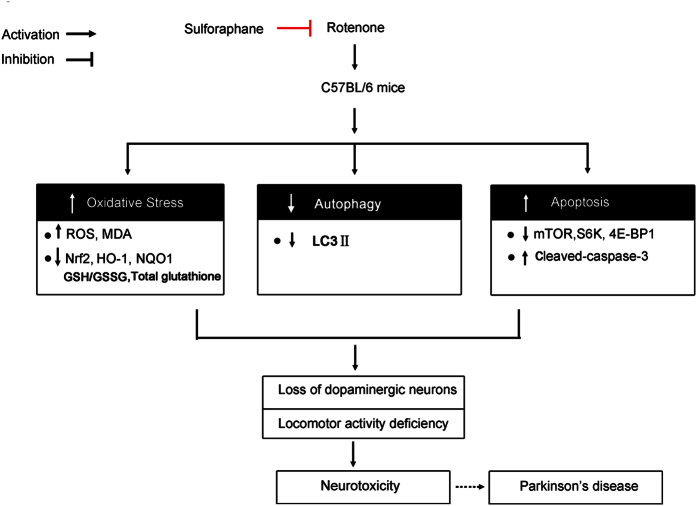
The possible molecular mechanism of sulforaphane-mediated neuroprotection against rotenone neurotoxicity *in vivo*.

The kinase mTOR is a major negative regulator of autophagy^57^. However, the interplay between mTOR and autophagy is complex in PD. For example, some studies found that rapamycin, an inhibitor of mTOR, protected against neuronal injury through induction of autophagy *in vivo*^16^,^58^,^59^. A similar scenario was also observed, in which rapamycin-activated autophagy inhibited rotenone-induced neuronal apoptosis^19^. However, one report showed that the autophagy process is altered independently of mTOR pathway disruption, and that the two inhibited could contribute to neuronal damage in PD^57^. Therefore, though alterations in mTOR signalling are linked to autophagy, the relationship between sulforaphane neuroprotection, mTOR signalling, and autophagy processes in PD does not seem mutually dependent. It has been shown that Nrf2 is related to mTOR, and our data supported the notion that sulforaphane exerts neuronal protective effects via activating Nrf2 and mTOR. This is consistent with a recent report that salvianolic acid A results in a dramatic activation of the Akt/mTOR signalling, followed by activation of the Nrf2/HO-1 axis in retinal pigment epithelia cells^60^. Understanding the interactions among these systems may be helpful in revealing the full mechanism of how sulforaphane suppresses rotenone-induced neurotoxicity. A summary of our findings is shown in Fig. 6.

## Additional Information

**How to cite this article**: Zhou, Q. *et al.* Sulforaphane protects against rotenone-induced neurotoxicity *in vivo*: Involvement of the mTOR, Nrf2, and autophagy pathways. *Sci. Rep.* **6**, 32206; doi: 10.1038/srep32206 (2016).

## Supplementary Material

Supplementary Information
