# Supplementary material for: Sulforaphane protects against rotenone-induced neurotoxicity in vivo: Involvement of the mTOR, Nrf2, and autophagy pathways
Source: Sci Rep. 2016 Aug 24;6:32206. doi: 10.1038/srep32206 (PMC4995453; doi:10.1038/srep32206)
Supplement: Supplementary Information [file srep32206-s1.pdf]

Sulforaphane protects against rotenone-induced neurotoxicity *in vivo*: Involvement of the mTOR, Nrf2, and autophagy pathways

Qian Zhou [1], Bin Chen [3], Xindong Wang [1], Lixin Wu [1], Yang Yang [1,2], Xiaolan Cheng [1,2], Zhengli Hu[1], Xueting Cai [1,2], Jie Yang [1,2], Xiaoyan Sun [1,2], Wuguang Lu [1,2], Huaijiang Yan [1,2], Jiao Chen[1,2], Juan Ye [1,2], Jianping Shen [1*], Peng Cao [1,2]

Supplementary Figure. 1

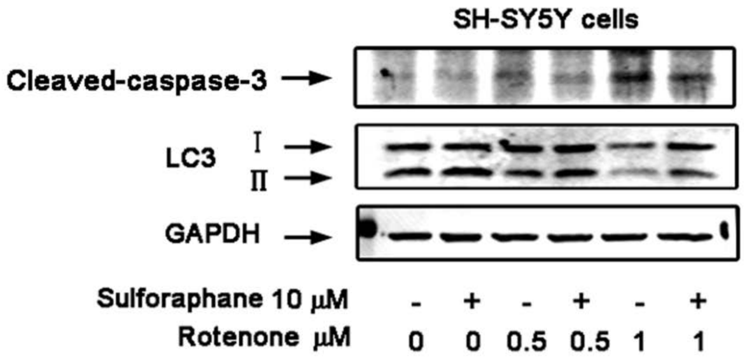

Sulforaphane protected against rotenone neurotoxicity via modulating autophagy *in vitro.* Representative immunoblots for LC3, cleaved-caspase-3, and GAPDH in SH-SY5Y cell line.
